# Antioxidant Property of Coffee Components: Assessment of Methods that Define Mechanisms of Action

**DOI:** 10.3390/molecules191119180

**Published:** 2014-11-19

**Authors:** Ningjian Liang, David D. Kitts

**Affiliations:** Food, Nutrition and Health, Faculty of Land and Food Systems, the University of British Columbia 2205 East Mall, Vancouver, BC V6T-1Z4, Canada; E-Mail: ningjian.liang@alumni.ubc.ca

**Keywords:** coffee, antioxidant, oxidative stress

## Abstract

Coffee is a rich source of dietary antioxidants, and this property, coupled with the fact that coffee is one of the world’s most popular beverages, has led to the understanding that coffee is a major contributor to dietary antioxidant intake. Brewed coffee is a complex food matrix with numerous phytochemical components that have antioxidant activity capable of scavenging free radicals, donating hydrogen and electrons, providing reducing activity and also acting as metal ion pro-oxidant chelators. More recent studies have shown that coffee components can trigger tissue antioxidant gene expression and protect against gastrointestinal oxidative stress. This paper will describe different *in vitro*, cell-free and cell-based assays that both characterize and compare the antioxidant capacity and mechanism of action of coffee and its bioactive constituents. Moreover, evidence of cellular antioxidant activity and correlated specific genomic events induced by coffee components, which are relevant to antioxidant function in both animal and human studies, will be discussed.

## 1. Introduction

Oxidative stress is a commonly used term to describe a disturbance in the balance between the production of free radicals that, upon accumulation, lead to the cellular breakdown of critical macromolecules and the status of *in vivo* antioxidant defense mechanisms that are required to combat the reactive nature of these molecules. Reactive oxygen species (ROS) derived from internal and external Fenton or Heiber Weiss reactions, including, for example, hydroxyl and superoxide anion radicals and hydrogen peroxide, are inactivated by enzymatic antioxidant systems, or peroxidases (e.g., salivary peroxidases and micro-peroxidases). The diet has a major role in contributing to both the source of free radicals, as well as combating the reactive nature of free radicals. The latter is accomplished by supplying specific substrates (e.g., glutathione) for PODs or, alternatively, by directly inactivating them. In general, different assays have been used to assess and compare the antioxidant activity of coffee according to the presence of a specific ROS in question [1].

**Figure 1:**
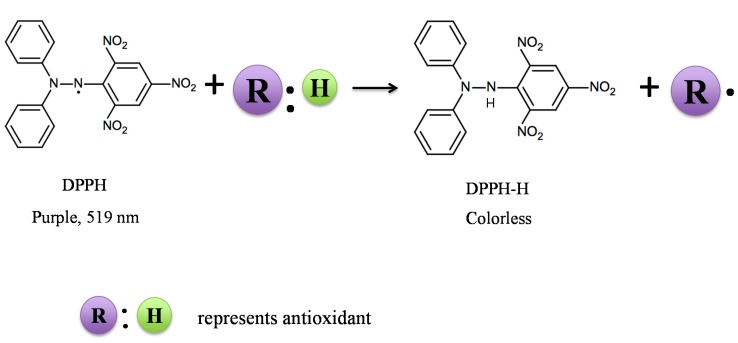
Reaction mechanism of 2,2-diphenyl-1-picrylhydrazyl (DPPH) with antioxidant. R:H = antioxidant radical scavenger; R = antioxidant radical.

Coffee is a very popular beverage consumed by many societies and a rich source of non-enzymatic, bioactive constituents, with noted antioxidant capacity. Thus, brewed coffee represent an important postprandial response to *in vivo* oxidative stress. Fogliano and Morales have reported global dietary intakes of coffee melanoidins that range from approximately 5 to 40 mg/kg/day in 28 different countries [2]. Coupled with the fact that as much as 25% of the antioxidant activity of melanoidins remains after 24 h of fermentation *in vitro* [3], this indicates that coffee should be recognized as a major source of dietary antioxidants that provide protection to the intestine during a normal gastrointestinal transit time. Evaluation of the antioxidant capacity of coffee therefore has been the focus for many studies that have used distinct *in vitro* chemical and enzymatic assays; some of which employ stable radicals as probes to quantitate free radical scavenging activity. Other assays to be described employ methods that generate non-stable reaction products to assess the radical quenching power of coffee constituents. Former, but still very popular methods are the chemical antioxidant assays that involve chromogen compounds of a radical nature used to simulate ROS. The presence of antioxidant compounds present in coffee leads to the disappearance of radical chromogens, and the activity in doing so is calculated from the disappearance of color or absorbance readings generated from a specific UV spectra. Examples of widely used chromogens that have received considerable use in chemical methods of antioxidant detection are the stable free radicals, DPPH and ABTS•^+^, in the DPPH and ABTS assay, respectively. In the DPPH assay, an odd electron displays a strong absorption band at a wavelength of 519 nm, which loses absorption once the odd electron is paired off by a hydrogen or electron-donating antioxidant (Figure 1). Other chemical assays use unstable free radicals, such as peroxyl radical (ROO^−^), superoxide radical anion (O^2−^) and hydroxyl radical (HO^−^) as examples of ROS, generated at fixed rates over time in chemical reactions that attempt to mimic a physiological mechanism *in vivo*. In these examples, the oxygen radical antioxidant capacity (ORAC), hydroxyl radical scavenging assay and superoxide radical scavenging capacity assay have all been used, respectively. DPPH, ABTS, FRAP, ORAC, hydroxyl radical scavenging assay and O^2−^ scavenging capacity assay have been used to measure the antioxidant activity of coffee beans/brew by different investigators. Finally, an important extension from the free radical scavenging power of coffee extracts is to extrapolate these results with potential anti-peroxidation activity, using model lipid systems and end-point measures of primary lipid oxidation [4]. Table 1 lists some examples of the application of these assays in evaluating the antioxidant activity of coffee beans/brew.

**Figure 2:**
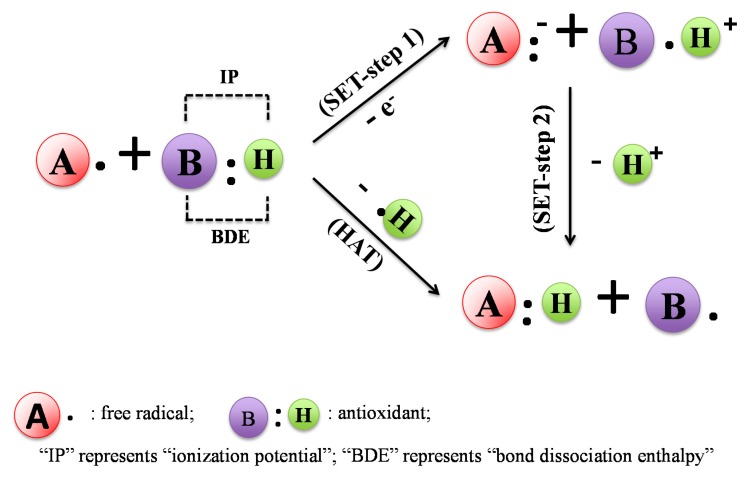
Mechanisms of antioxidant reacting with free radical: single electron transfer (SET) and hydrogen atom abstraction (HAT). In SET mechanism, the IP (ionization potential) of the antioxidant is the most important energetic factor in evaluating the antioxidant action. In the HAT mechanism, the BDE (bond dissociation enthalpy) of the antioxidant is the important parameter in evaluating the antioxidant action.

Antioxidants react with free radicals by different mechanisms—hydrogen atom transfer (HAT) or single electron transfer mechanism (SET); or the combination of both HAT and SET mechanisms being primary examples. The HAT reaction is a concerted movement of a proton and an electron in a single kinetic step, as shown in Figure 2. In HAT mechanisms, the free radical removes one hydrogen atom of antioxidant, and the antioxidant itself becomes a radical. In this mechanism, the bond dissociation enthalpy (BDE) is an important parameter in evaluating the antioxidant action [5]. The lower the BDE of the H-donating group in the potential antioxidant, the easier it will be for the reaction of free radical inactivation. The SET reaction is imitated by single-electron transfer from the nucleophile to the substrate, producing a radical intermediate, whose fate can then be involved in any number of events, as shown in Figure 2. In SET mechanisms, the antioxidant provides an electron to the free radical and itself then becomes a radical cation. In this mechanism, the ionization potential (IP) of the antioxidant is the most important energetic factor in evaluating the antioxidant action. The lower the ionization potential, the easier is the electron abstraction [6]. It is very difficult to distinguish between HAT and SET reactions. In most situations, these two reactions take place simultaneously, and the mechanism of the reaction is determined by the antioxidant’s structure and solubility, the partition coefficient and solvent polarity. Examples of HAT-based assays include ABTS and ORAC. Examples of SET-based assays include DPPH and FRAP [7].

**Table 1:** Mechanisms of chemical assays commonly used to evaluate the antioxidant capacity of coffee and examples of the application of these assays to measure the antioxidant activity of coffee beans/drinks. SET, single electron transfer; HAT, hydrogen atom transfer; AAPH, 2,2'-azobis(2-amidinopropane) dihydrochloride; XOD, xanthine oxidase; NBT, nitro blue tetrazolium.

Assay	Source of Free Radical	Reaction Mechanisms	Method of Quantification of the Targeted Free Radical	Application to Measure Antioxidant Activity of Coffee		
				Species of the Beans	Roasting Degree of the Beans	Ref.
DPPH	Dissolve DPPH in ethanol	SET or HAT	Measure the absorption at 517 nm; EPR (please define)	Blend of different varieties	Green	[8]
				Blend of different varieties	Light, medium and dark	[9]
				*Arabica* and *Robusta*	Green	[10]
				*Arabica* and *Robusta*	*Arabica* (219 °C for 905 s); *Robusta* (228 °C for 859 s)	[11]
ABTS	Oxidize ABTS with potassium persulfate	HAT	Measure the absorption at 645 nm, 734 nm or 815 nm; EPR	Blend of 80% *Arabica* and 20% *Robusta*	Green, light, medium and dark	[12]
				*Arabica*, *Robusta* and a blend of these two	Green, medium and dark	[13]
				*Arabica*	Light (225 °C for 3 min); medium (233 °C for 3 min); dark (240 °C for 3 min)	[14]
				*Arabica* and *Robusta*	*Arabica* (219 °C for 905 s); *Robusta* (228 °C for 859 s)	[11]
FRAP	Fe^3+^/tripyridyltriazine complex	SET	Measure the absorption of ferrous at 593 nm	Blend of different species	Green	[8]
				*Arabica*	Medium, dark and blend of medium (70%) and dark (30%)	[15]
				*Arabica* and *Robusta*	Green	[10]
ORAC	Dissolve AAPH in buffer to form peroxyl radicals	HAT	β-phycoerythrin Fluorescein	*Arabica*	Not declared	[16]
				*Arabica* and *Robusta*	Light, medium, and dark	[17]
				*Arabica*	Green	[18]
HO^−^ Scavenging Assay	Fe^2+^ + H_2_O_2_ DMSO + H_2_O_2_ Photochemical decomposition of H_2_O_2_	Not defined	Deoxyribose, Benzoate salicylate EPR with spin trap reagent (DMPO)	*Arabica* and *Robusta*	Green	[10]
				Not declared	Not declared	[19]
				*Arabica*	Green	[18]
				*Arabica* and *Robusta*	190 ± 3 °C for 18–20 min	[20]
O^2−^ Scavenging Capacity Assay	Hypoxanthine/xanthine + O_2_ + XOD→O^2−^ + uric acid PMS + NADH→O^2−^	Not defined	O^2−^ reduce the probe NBT to a purple formazan that could be measured at 562 nm EPR with spin trap reagent (DMPO or BMPO)	*Arabica*	Green	[18]

## 2. *In Vitro* Assays Commonly Used to Evaluate Antioxidant Activity of Coffee and Mechanisms of Action

### 2.1. DPPH Assay

2,2-diphenyl-1-picrylhydrazyl (DPPH), is a stable free radical with an unpaired electron that is delocalized over the entire molecule [21] and, thus, employed in the DPPH assay. DPPH possesses a purple color, with a maximum absorption at 519 nm in ethanol; hence, scavenging the DPPH radical by coffee antioxidants will result in a decrease in absorption readings over time; the extent of decrease in DPPH absorption being proportional to the concentration of radicals that are being scavenged, according to the principle of Blois [22]. Measurements are made using a UV-visible spectrophotometer at room temperature, and the scavenging capacity is represented as the percentage of DPPH radical inhibition. The DPPH assay is based on both electron transfer (SET) and hydrogen atom transfer (HAT) reactions [23]. An advantage of the DPPH assay is that it is an easy, economic and rapid method to evaluate the radical scavenging activity of non-enzymatic antioxidants [22]. Since DPPH is a stable radical, this assay considers not only the concentration of the tested sample, but also the reaction time and the temperature; both of which when controlled carefully enable this assay to be highly reproducible. There are, however, limitations to this assay when used to measure the antioxidant activity of brewed coffee are related to the color of the coffee, thus potentially interfering with the DPPH absorption. Furthermore, DPPH is a lipophilic radical with limited accessibility to the hydrophilic components present in brewed coffee, thereby requiring alcohol in the reaction mixture to ensure maximum solubility. The presence of ethanol adds to a background antioxidant activity, which needs to be considered when standards are constructed for quantification purposes. Proteins, if present in the brewed coffee will also interfere with the assay once precipitated by the presence of ethanol in the reaction system. The biggest limitation of the DPPH assay is that it is not related to specific free radicals that have physiological relevance.

### 2.2. ABTS Assay

The ABTS assay utilizes a free radical, mono-cation of 2,2'-azino-bis 3-ethylbenzothiazoline-6-sulphonic acid) (ABTS), which is generated when ABTS substrate is oxidized with potassium persulfate. ABTS•^+^ has a blue/green color with maximum absorption spectra at 734 nm, in water. The more hydrophilic free radical, namely the pre-generated ABTS•^+^, is decolorized when reduced in the presence of the test sample. This event indicates the extent of relative radical scavenging activity expressed as a percent inhibition. Alternatively, this chemical response can also be compared to a water-soluble vitamin E analogue, Trolox (6-hydroxy-2,5,7,8-tetramethychroman-2-carboxylic acid) when tested under the identical conditions [24]. The results are then expressed as TEAC (Trolox equivalent antioxidant capacity). The results of the ABTS assay should be comparable to results found in the DPPH assay and may be viewed as confirmation of the DDPH assay, albeit that absolute values from the ABTS assay are generally higher [25]. Both radicals show the same stoichiometry with water-soluble vitamin E analogue, Trolox (e.g., two moles of ABTS•^+^ [26] or two moles of DPPH radicals [27] are scavenged by one mole of Trolox). However, ABTS has an advantage to the DPPH assay, since ABTS can be used at different pH values and therefore takes into consideration the effect of pH on the antioxidant activity of the tested sample [28]. The ABTS assay is also applicable to testing antioxidant compounds that have a lipophilic or hydrophilic character [24], which is particularly important when testing different coffee varieties or isolated fractions collected from brewed coffee. The limitation of the ABTS assay, however, involves both possible color interference from the coffee browning pigments present in the brew and, as is the case with the DPPH assay, produces results that have little physiological relevance to naturally produced unstable radicals. In general, highly pigmented and hydrophilic antioxidants are better assessed for antioxidant activity using the ABTS assay compared to the DPPH assay [29]. Since roasted coffee is rich in pigments and possesses hydrophilic components in a complex matrix derived from both brewed coffee [30] and extracts recovered from spent coffee [11], the ABTS assay enables more complete information on the relative antioxidant capacity of different coffees or related bioactive constituents.

### 2.3. FRAP and TRAP Assays

The ferric ion reducing antioxidant power (FRAP) assay is a non-specific, redox-linked, colorimetric assay that is related to the molar concentration of the antioxidant present. Since FRAP measures the ferric reducing activity of the sample, a low pH buffer is required to reduce the ferric tripyridyltriazine complex (Fe^3+^-TPTZ) to a ferrous (Fe^2+^-TPTZ) form, which is quantitated at an absorption maximum of 593 nm [31]. The increase in absorbance at 593 nm is proportional to the total ferric reducing power of the tested sample. Results are presented as mg of Fe^2+^/g of dried sample and represent the mass of Fe^3+^, which can be reduced by 1 g of dried sample. In most systems, the condition of the assay favors the reduction of the Fe^3+^-TPTZ complex and, thus, color development, to the degree to which antioxidant activity is present. Thus, there is an excess amount of Fe^3+^ used in the assay, and therefore, the rate-limiting factor for the Fe^2+^-TPTZ is the reducing activity of the sample. FRAP has been automated to give results within 10 min using the change in absorbance (ΔA_593nm_) from a reagent blank; the final calculation for each sample being associated with a (ΔA_593nm_) of a Fe^2+^ standard. Although the FRAP assay was originally developed to measure the antioxidant power of plasma, this simple, highly reproducible and inexpensive assay has also been used to assess the total antioxidant capacity of different food systems, including coffee [31]. When interpreting the results of this test, it is important to understand that this assay measures the capacity of a sample to participate in one-electron redox reactions; hence, other antioxidant compounds present in the coffee, which may possess different modes of action (e.g., radical scavengers), will not be included in the reaction. Although the assay is compatible with coffee beverages, which contain mostly water-soluble components that react in the aqueous solution, the reducing capacity is determined in the absence of molecules that may have protective capacities to detoxify reactive oxygen species. For example, nitric oxide when reacting with O^2−^ will produce a highly reactive peroxynitrate radical (ONOO^−^), which, in turn, can damage important biomolecules. It is not always possible to attribute NO scavenging power to any particular class of coffee bioactive compounds in this assay. Finally, FRAP does not measure potentially important coffee antioxidant components that contain thiol groups, due to a redox potential threshold that is below FRAP detection [32]. Some examples of sulphur containing compounds present in coffee that exhibit antioxidant activity include thiazole and related derivatives [33]. Moreover, non-activity interaction has been reported between different antioxidants when FRAP is used [34]. FRAP, and other SET-based assays, have in common a limitation that they only reflect reducing capacity and, therefore, do not identify potential antioxidants that work through a HAT mechanism. Since most phenolic compounds, such as chlorogenic acid in coffee, exhibit antioxidant activity by hydrogen donation [35], SET-based assays, such as DPPH and FRAP, are limited in quantitatively describing the antioxidant activity of coffee beverages.

Unlike the FRAP assay, the TRAP (total peroxyl radical-trapping antioxidant parameter) is based on the application of the thermal decomposition of water-soluble, azo-compound 2,2'-azobis-(2-amidinopropane hydrochloride) to peroxyl radicals [36]. Spectra are recorded over a 10–15 min period at frequent (90 s) intervals, and the absorbance at 734 nm is measured as a function of time. In all of these assays, the unit of activity can be expressed as a Trolox equivalent antioxidant capacity (TEAC) or defined as the concentration (mM) of Trolox having equivalent antioxidant capacity to a 1.0 mM solution of the substance under study. TEAC values derived from different antioxidant components, such as vitamin C and vitamin E, are considerably different, thus this assay has severe limitations to account for the antioxidant capacity of coffee beverages, since they represent relatively complex mixtures of individual components that share antioxidant activity.

### 2.4. ORAC Assay

The oxygen radical antioxidant capacity (ORAC) assay is used to evaluate the capacity of antioxidant compounds that scavenge peroxyl radicals generated by 2,2'-azobis(2-amidinopropane) dihydrochloride (AAPH). This method, originally described by Cao and others [37], is a popularly used HAT method to evaluate model Maillard reactions [38] and coffee constituents [39]. The assay originally used β-phycoerythrin as an indicator protein and AAPH as a peroxyl radical generator. AAPH constantly generates peroxyl radicals, which, in turn, oxidizes β-phycoerythrin, thus reducing the fluorescence intensity of β-phycoerythrin. The assay therefore measures the extent to which the antioxidant sample protects the β-phycoerythrin from oxidation in the presence of AAPH. The protective effect of the tested sample is measured from the area under the test sample fluorescence decay curve, as compared to that of the blank. β-phycoerythrin has a limitation due to nonspecific binding with polyphenols; hence, Ou and others replaced β-phycoerythrin with fluorescein [40] in a modified ORAC assay that evaluates more accurately the peroxyl radical scavenging capacities of aqueous soluble components present in coffee [17,41]. ORAC assay is not only suitable for measuring the antioxidant capacity of the hydrophilic components in brewed coffee, but can be adjusted to determine the antioxidant capacity of hydrophobic components by changing the buffer system [42]. This advantage enables quantitation of antioxidant capacity for both hydrophilic and hydrophobic components in coffee. In addition, the assay, which requires the area under the fluorescence decay curve to be determined at a fixed time period, actually reflects the anti-peroxyl radical capacity of antioxidants that may have different free radical scavenging rates and reaction kinetics. ORAC is therefore suitable to study the antioxidant capacity of brewed coffee, which is a complex mixture of many components having different reaction kinetics. Extrapolation of ORAC results to whole body redox status is limited by the fact that both the oxidation rate and characteristics of fluorescein in the presence of peroxyl radical only mimic the reaction subtracts *in vivo*.

### 2.5. Hydroxyl Radical Scavenging Assay

The hydroxyl radical is one of the most reactive free radicals in a biological system. Therefore, it is important to consider the hydroxyl radical scavenging capacity of dietary antioxidants. Hydroxyl radicals can be generated by Fenton reaction between ferrous iron and H_2_O_2_ [43]. Hydroxyl radicals are also generated by the reaction between DMSO and H_2_O_2_ [44]. Different probes, such as deoxyribose [45], benzoate [45] and salicylate [46], have employed colorimetric or fluorometric measures to indicate the damage caused by hydroxyl radicals. PM2 bacteriophage DNA has also been used to show the protection of coffee constituents against hydroxyl radical-induced single- and double-strand DNA scission [47]. Hydroxyl radical scavengers present in tested samples protect the probe from being damaged by hydroxyl radicals. The percentage of hydroxyl radical scavenging activity of test sample is determined in comparison with a negative control. In addition to using probes, hydroxyl radical can also be quantified by electron paramagnetic resonance (EPR) with the help of a spin-trap agent. Spin trapping is a technique where a nitrone- or nitroso- compound reacts with a target free radical to form a relatively stable adduct, which is measurable with EPR spectroscopy and gives a distinguishable EPR spectrum. For example, 5,5-dimethyl-1-pyrroline N-oxide (DMPO) is commonly used to trap short-lived hydroxyl radicals to form relatively stable DMPO-OH adducts that are detected and quantified by EPR [43]. However, attention is needed to consider the stability of the DMPO-OH adduct in the presence of Fe^2+^ ions, because Fe^2+^ ions can quench the DMPO-OH radical quite rapidly [48]. Therefore, the selection of the appropriate spin trap is critical to form stable spin-adducts in specific environments that define different reaction systems. The hydroxyl radical scavenging capacity assay has been used to study green coffee [10]. A potential limitation of this assay for measuring the antioxidant activity of coffee is that coffee components can chelate transition metal ions, such as Fe^2+^ and, consequently, interfere with the Fenton reaction, which normally generates the hydroxyl radical. Therefore, it is difficult to characterize the antioxidant activity of coffee components; whether they are scavenging the hydroxyl radical directly or acting indirectly by chelating Fe^2+^. Another limitation of this procedure has to do with the fact that roasted coffee beans and brewed coffee contain H_2_O_2_, a precursor component of the Fenton reaction.

### 2.6. O^2−^ Scavenging Capacity Assay

The O^2−^ scavenging capacity assay involves using a superoxide radical anion (O^2−^) that is generated through either enzymatic or non-enzymatic O^2−^ reaction systems. In the enzymatic system, xanthine oxidase (XOD) utilizes hypoxanthine or xanthine as the substrate and O_2_ as a cofactor to produce O^2−^ and uric acid [49]. The generation of O^2−^ *in situ*, by electron transfer from NADH to O_2_ present in solution, is analogous to NADPH oxidase- and NADH dehydrogenase-catalyzed generation *in vivo*. For a non-enzymatic system, phenazine methosulfate is used to oxidize nicotinamide adenine dinucleotide to generate O^2−^, nitro blue tetrazolium (NBT) is used as the probe to quantitate the concentration of O^2−^ [50]. O^2−^ reduces NBT to a purple formazan. After incubation of tested samples with the phenazine methosulfate-NADH-NBT mixture, the absorbance at 562 nm is measured against a blank to determine the degree of O^2−^ scavenging. EPR spectroscopy is a well-defined alternative method to measure superoxide radical concentration. Similar to hydroxyl radical, O^2−^ is a short-lived free radical and requires DMPO to form a DMPO-OOH adduct with O^2−^, which can then be detected by EPR spectroscopy. The limitation of DMPO is that it does not distinguish between superoxide and hydroxyl radicals due to the spontaneous decay of DMOP-OOH adduct into DMPO-OH adduct. As a result, other spin traps, such as BMPO, are used to extend the half time without worrying about fast decomposition from BMOP-OOH to BMOP-OH. The superoxide radical scavenging capacity of selected coffee varieties has been evaluated [1]. O^2−^ is poorly reactive and can easily decompose to form more potent and reactive free radical species, such as hydroxyl radical (HO^−^) and peroxynitrite (ONOO^−^). Moreover, constituents that are pigmented or have fluorescence in the coffee sample might further interfere with the colorimetric or fluorometric responses. Using EPR spectroscopy with an appropriate spin trap agent yields more reliable results than using fluorometric probes to measure the level of O^2−^ after reacting with/without coffee beverage.

## 3. Coffee Components with Antioxidant Activity (Chemical Assays)

Green coffee beans are a complex source of multiple bioactive constituents with characteristic free radical or antioxidant activity, which include, in varying quantities, caffeine, chlorogenic acid, trigonelline, cafestol and kahweol, depending on the source. In addition, the process of roasting coffee beans produces a series of changes to the chemical composition of coffee, leading to the formation of the characteristic flavor, aroma and browning pigments. In roasted coffee beans, melanoidins generated from non-enzymatic browning exhibit antioxidant activity. Therefore, the antioxidant capacity of brewed coffee not only is contributed to by those components originally present in green beans, but also by the components that are generated during the conditions of the roasting process. The antioxidant capacities of hydrophilic coffee components, such as caffeine, chlorogenic acid and melanoidins, against different free radicals have been extensively investigated using different chemical assays. The studies on the antioxidant activity of hydrophobic compounds, such as cafestol, kahweol and trigonelline, are focused on biological systems that include cell culture, as well as animal and human clinical trials.

### 3.1. Caffeine

The caffeine content in coffee beans ranges between 10 and 12 mg/g in *Arabica* beans [51] and 19–21 mg/g in *Robusta* beans [52] and changes only slightly during the roasting process at different temperatures [52]. The caffeine content in coffee beverage also depends on the coffee brewing method or prior processing; for example, caffeine content in boiled coffee (~90 mg/100 mL) is higher compared to that found in filtered coffee (~120 mg/100 mL) [53]. Caffeine content in different brewed coffees ranges considerably from espresso coffee, which contains 51–322 mg/180 mL serving size, to decaffeinated coffee, where the range is 0–13.9 mg/serving size [54]. Although caffeine is well known for producing bioactive symptoms that include the reduction of fatigue and enhancing alertness, it also has an antioxidant property, as shown by the affinity to scavenge hydroxyl radical [55] with bimolecular rate constants that range from 2.6 × 10^9^ M^−1^s^−1^ [19] to 5.9 × 10^9^ M^−1^s^−1^ [56]. In these studies, HO^−^ generated by the Fenton reaction [56], or photochemical decomposition of hydrogen peroxide [19], and the HO^−^ scavenging activity by caffeine were measured using EPR, with the assistance of the spin trap technique. Caffeine has also been shown to scavenge superoxide radical with a bimolecular rate constant of 7.5 × 10^1^ M^−1^s^−1^ [57]. In this study, xanthine and xanthine oxidase were the source of O^2−^, and EPR was used to measure the concentration of O^2−^ after reacting with caffeine. Other studies attempted to demonstrate the ROO^−^ scavenging activity of caffeine using the ORAC assay; however, no activity was observed at the micromolar level [58,59]. This was in contrast to the metabolites of caffeine (e.g., 1-methylxanthine and 1-methyluric acid), where significant ROO^−^ scavenging capacity was observed at a micromolar concentration [58].

### 3.2. Chlorogenic Acids

Chlorogenic acids (CGAs) consist of a family of esters formed between trans-cinnamic acids, such as caffeic acid and ferulic acid, with quinic acid [60]. The ester linkage is formed between the carboxyl group of trans-cinnamic acids and all four of the quinic acid hydroxyl groups. Subclasses of CGAs include caffeoylquinic (CQA), feruloylquinic (FQA) and dicaffeoylquinic (diCQA) acids. Three isomers exist in each subclass, yielding nine CGAs in total, with 5-O-caffeoylquinic acid (5-CQA) being the dominant form present in coffee beans. A regular cup of coffee contains between 20 to 675 mg CGAs, depending on the source of the coffee beans, the roasting temperatures used and the methods of brewing [61,62]. For example, one cup of *Arabica* coffee delivers between 70 and 200 mg of CGAs, whereas *Robusta* coffee contains between 70 and 300 mg of CGAs [61]. CGAs are polyphenols with known antioxidant activity, and 5-CQA is a powerful hydroxyl radical scavenger with a HO^−^ scavenging rate constant of 7.73 × 10^9^ M^−1^s^−1^ [63]. This result was determined using the Fenton reaction to generate HO^−^ [56], and when in the presence of 5-CQA, the scavenging rate constant could be measured using EPR with a spin trap. 5-CQA is also an effective superoxide radical scavenger in the xanthine/XOD, O^2−^ generating system [64], with NBT as the probe to indicate the amount of O^2−^ after reacting with 5-CQA. The peroxyl radical scavenging ability of 5-CQA is approximately 12 mmol Trolox equivalent/g [65]. Other isomers of CGAs are believed to be effective antioxidants. For example, Xu *et al.* [66] compared the antioxidant capacity of diCQA with CQA and reported that diCQA possessed a relatively stronger scavenging capacity when measured using ABTS•^+^ and DPPH radicals. This finding was attributed to the greater number of available hydroxyl groups in diCQA compared to CQA.

### 3.3. Coffee Maillard Reaction Products

Maillard reaction products (MRP) that provide the aroma, flavor and color of different brewed coffees are generated during the roasting process and can also contribute to the antioxidant activity of this beverage through both direct free radical scavenging activities, as well as metal pro-oxidant sequestering activity [67,68,69]. Coffee melanoidins are high molecular weight, brown colored, polymeric substances that are formed during the Maillard reaction with coffee roasting; contributing up to 25% of the brewed coffee dry matter [70]. Although some known phenolic antioxidants, such as chlorogenic acid in coffee, are lost during the roasting process, the formation of melanoidins appears to compensate for any possible reduction in the level of antioxidant activity. The structures of coffee melanoidins are not fully elucidated because many constituents, such as polysaccharides, proteins [71] and phenolics, are modified to lesser known structures during coffee melanoidin formation [72]. In order to study the antioxidant activity of coffee melanoidins, different protocols, including gel filtration chromatography [70], ultrafiltration [73] and hydrophobic interaction chromatography [71], have been used to isolate specific melanoidin fractions for subsequent antioxidant testing, using a number of different photometrical methods that include DPPH, ABTS and ORAC assays, respectively. The mechanisms of antioxidant action of melanoidins has been attributed to affinities that include scavenging hydroxyl and proxy radicals, respectively, breaking the radical chain by donation of hydrogen and also to chelating pro-oxidant transition metal ions [12]. The hydroxyl radical scavenging capacity of melanoidins was shown with hydroxyl radicals generated by the Fenton reaction, which exhibited reduced capacity to degrade deoxyribose in the non-specific deoxyribose antioxidant assay [74]. Purifying melanoidins, such as those recovered in 2 M NaCl, resulted in a 50% loss of peroxyl radical scavenging capacity, compared to that before purification [75]. Hence, the presence of low molecular weight compounds linked non-covalently to the melanoidin skeleton contributes significantly to the observed peroxyl radical scavenging capacity attributed to coffee melanoidins [69]. The antioxidant activity of coffee melanoidins after *in vitro* enzymatic digestion has also been evaluated using DPPH, ABTS, FRAP and ORAC assays, and the findings indicate that low molecular weight compounds bound to melanoidins after digestion exert the highest relative antioxidant activity [76].

In addition to melanoidins, a number of heterocyclic compounds, including 1-methylpyrrole and derivatives, as well as thiazole and thiazole derivatives, have been identified in brewed coffee. These constituents were effective at inhibiting heat-induced hexanol oxidation, a primary lipid oxidation product, thus confirming anti-peroxidation activity [33]. Pyrrole and related compounds have also been shown to react with hydroxyl radicals using EPR analysis [77]. The relative antioxidant activity of components in coffee was not as strong as synthetic antioxidant butylated hydroxytoluene (BHT).

## 4. Coffee Components with Antioxidant Capacity (Cellular Antioxidant Activity Assays and Animal Studies)

Although chemical-based antioxidant assays are useful for screening food constituents for antioxidant activity, the results cannot be totally extrapolated to biological systems, because antioxidant capacity is not limited to only free radical scavenging ability and reducing capacity, but also includes the activation of redox transcription factors and upregulation of genes that induce the expression of antioxidant enzymes. Cellular antioxidant activity assays provide biologically relevant methods to measure the activity of antioxidants at the cellular level, because they account for important factors, such as cellular uptake, distribution and metabolism [78]. To show the antioxidant activity of coffee components in cell-based model systems, investigators have used a number of different cell lines, stimulators of oxidative stress and various endpoint measures that point to specific underlying mechanisms of antioxidant activity or oxidative stress. For example, human intestine-derived Caco-2 cells [79], human liver-derived HepG2 cells [80], human vascular endothelia cell line EA. Hy926 [81] and human macrophage cell line U937 [82] have all been used in cell-based antioxidant mechanism assays. The oxidative stress in these examples has been induced by different stressors, such as chemical (AAPH, hydrogen peroxide and *tert-*Butyl hydroperoxide (t-BOOH)) and also physical (radiation and hyperoxia) stimulation. For example, the redox sensor, dihydrodichlorofluorescein, DCFH_2_, is commonly used as a probe to indicate the oxidative status in AAPH stimulated cells, because DCFH_2_ is easily oxidized to fluorescent dichlorofluorescein DCF by the peroxyl radicals generated from AAPH. The stronger the antioxidant activity of the tested sample, the less available the peroxyl radical; the consequence is a weaker fluorescent signal. The application of DCFH_2_ is not limited to AAPH stimulated peroxyl radical, but can also be used with H_2_O_2_ stimulation [80]. Actual measures of cellular metabolism resulting in changes in oxidative stress include determining the concentrations of reduced glutathione (GSH), the presence of a secondary lipid oxidation product, malondialdehyde (MDA), and activities of antioxidant enzymes glutathione peroxidase (GPx), glutathione reductase (GR) and superoxide dismutase (SOD). GSH is an important intracellular antioxidant that prevents damage from cellular ROS, as well as being a substrate for glutathione enzymes. MDA, a product of secondary lipid peroxidation, is a useful marker for indicating the extent of lipid oxidation that defines potential subsequent reactions with macromolecules and secondary oxidation products. For example, MDA reacts with DNA to form adducts that represent oxidative stress specific to DNA damage. An important measurement for this event involves detecting oxidized purines, such as 8-hydroxydeoxyguanosine [83].

Oxidative stress related transcription factors and antioxidant enzymes are also commonly used measures that reflect the redox status of the cell. Nuclear factor-E2-related factor 2 (Nrf2) is a key transcription factor that regulates the expression of numerous detoxifying and antioxidant enzymes that facilitate the cellular adaptation to oxidative stresses and possibly mitigating inflammatory responses [84]. Therefore, the level of Nrf2 that translocates to the nucleus is a useful indicator to show whether the tested sample has an antioxidant effect. Coffee constituents are demonstrated as modulators of Nrf2 nuclear translocation; thus, inducing an antioxidant response element-mediated expression of Phase II enzymes can involve detoxification and antioxidant defense [38,85].

Animal studies have also provided a comprehensive understanding on the ability of coffee constituents to modulate oxidative status *in vivo*. Since oxidative stress is the initiator of many chronic diseases, such as inflammation, diabetes, Parkinson’s disease and Alzheimer’s disease, different animal models have been employed to study the specific affinity of coffee to mitigate oxidative stress related to these disorders. Oxidative status in these animal models is determined by measuring the susceptibility of inducing lipid peroxidation, changes in antioxidant enzyme activity and upregulation of oxidative stress-related genes and transcription factors. Table 2 is a summary of the results and conclusions of the cellular and animal studies reviewed in this section.

### 4.1. Caffeine

The presence of caffeine in coffee reduces oxidative stress in hypoxia-induced pulmonary epithelial cells in a concentration-specific manner [86]. Caffeine has also been shown to be an inhibitor of hydrogen peroxide-induced lipid peroxidation products in human skin fibroblasts [87]. Chronic coffee and caffeine ingestion reduces lipid peroxidation in brain membranes of healthy rats with parallel increases in GSH concentration. This finding suggested that long-term coffee and caffeine ingestion has an important role in preventing age-associated decline in cognitive function through the protection of the antioxidant system and regulation of oxidative stress [88]. In a mice model with alcohol-induced liver damage, caffeine consumption decreased serum and tissue inflammatory cytokines levels and was active at reducing tissue lipid peroxidation and ROS [89]. The protective effect of caffeine against oxidative stress was also demonstrated in Alzheimer’s disease-like pathology in rabbit hippocampus induced by a cholesterol-enriched diet [90].

### 4.2. Chlorogenic Acid

5-CQA has been shown in numerous cell-specific model systems to protect against UVB- [91], hydrogen peroxide- [92], t-BOOH-, H_2_O_2_- and FeSO_4_- induced [93] oxidative stress by lowering lipid peroxidation and the loss of GSH, as well as upregulating intrinsic pathways that involve genes from the FOXO family [93]. Similar results were obtained in t-BOOH-stimulated human hepatoma HepG2 cells [94]. A recent study also demonstrated that CGA present in coffee induces phase II-enzymes via the Nrf2 pathway *in vitro* and *in vivo* [95]. In whole animal studies, CGA protected against oxidative stress triggered by the herbicide, paraquat [96], and a high fat diet/streptozotocin treatment [97].

**Table 2:** Summary of cell-based and animal-based studies on evaluating the effects of coffee components on modulating oxidative status.

Coffee Components	Cell-Based Studies				Animal-Based Studies			
	Cell Line	Oxidative Stress Stimulator	Description of Oxidative Stress after Coffee Component Treatment, Compared to the Negative Control	Ref.	Animal	Oxidative Stress Stimulator	Description of Oxidative Status after Coffee Component Treatment Compared to the Negative Control	Ref.
**Caffeine**	Pulmonary epithelial A549 cell	hyperoxia	↓ROS level	[86]	Rat	None	↑GR, ↑GSH, ↑SOD, (No change) in GPx in brain	[88]
	MLE 12	hyperoxia	↓ROS level	[86]	Mice	5% ethanol in diet	↓ROS, ↓TNF-α,↓ proinflammatory cytokines and chemokines in liver	[89]
	Human skin fibroblast WS-1 cell	H_2_O_2_	↓ROS level; ↓4-hydroxy-2-nonenal	[87]	Rabbit	Cholesterol-enriched diet	↑GSH, ↓ROS, ↓8-Isoprostaglandin F2α	[90]
**Chlorogenic acid**	Human HaCaT keratinocyte	UVB irradiation	↓DNA damage, ↓Apoptotic bodies, ↓Apoptosis-related proteins, ↑Cell viability	[91]	Rat	Paraquat	↑Liver triacylglycerol, ↑Phospholipid	[96]
	Mesenchymal stem cell	H_2_O_2_	↑Expression of FOXO family genes, ↓Apoptosis	[92]	Rat	High fat diet/streptozotocin treated	↓Thiobarbituric acid, ↑SOD, ↑Catalase	[97]
	PC12 cell	t-BOOH, or H_2_O_2_, or FeSO4	↑GSH, ↓MDA	[93]				
	Human hepatoma HepG2 cell	t-BOOH	↑GSH	[94]				
**Melanoidins**	Human hepatoma HepG2 cell	t-BOOH	↑GSH, ↓MDA	[98]	Rat	High-fat, high-calorie solid diet	↓Pro-inflammatory cytokines, ↑Anti-inflammatory cytokines	[99]
	Human neuroblastoma cell IMR32	H_2_O_2_	↑Cell viability	[100]				
**Trigonelline**	Not available				Rat	Streptozotocin treated	↑SOD, ↑Catalase, ↑GSH, ↓MDA, ↓NO	[101]
**Cafestol and kahweol**	Neuronal cell line SH-SY5Y	6-Hydroxydopamine	↑Nrf2 nuclear translocation,	[102]	Mice	CCl_4_	↑GSH, ↓MDA	[103]
	NIH3T3 cell	H_2_O_2_	↓ TBARS, ↓ ROS, ↓ DNA damage	[104]				

### 4.3. Coffee Maillard Reaction Products

Similar findings exist with digested coffee melanoidins at physiological concentrations with respect to the affinity to protect against t-BOOH-induced oxidative stress, as indexed by the increased cellular concentration of GSH and lower MDA in human hepatoma HepG2 cells [98]. Melanoidins with molecular weights greater than 3.5 kDa, recovered from brewed dark roasted coffee, increased the survival rate of human neuroblastoma cells IMR32 pretreated with H_2_O_2_ [100]. The protective effect of high molecular coffee melanoidins (>12 kDa) against liver oxidative stress has also been studied in rats fed high-fat, high-calorie, solid diets. In this study, quantitating the reduction in liver pro-inflammation was achieved by examining the corresponding elevation of specific anti-inflammatory cytokine biomarkers [99].

### 4.4. Trigonelline

The antioxidant activity of trigonelline has not been evaluated in specific cell models, but has been shown to reduce oxidative stress in diabetic rats through an upregulation of antioxidant enzyme activity and a decrease in lipid peroxidation [101]. *N*-methylpyridinium is a thermal degradation product of trigonelline during the coffee roasting process [105]. *N*-methylpyridinium is a bioactive that has activity to induce Nrf2 antioxidant-response element gene transcription [85]. Feeding of *N*-methylpyridinium also resulted in an increased total antioxidant capacity in the plasma of rats, which was assessed by measuring the inhibitory effect on linoleic acid, an anti-peroxidation property, and expressing the result as Trolox equivalent [106].

### 4.5. Cafestol and Kahweol

Cafestol and kahweol are two diterpenes present in coffee beans, recovered from unfiltered coffee beverage. Both have shown antioxidant activity in cell models and mice models that involved triggering the upregulation of key antioxidant enzymes. Kahweol was effective at protecting neuronal cells, SH-SY5Y, from oxidative stress induced by the Parkinson’s disease-related neurotoxin, 6-hydroxydopamine, by inducing Nrf2 nuclear translocation [102]. A mixture of cafestol and kahweol has also been shown to reduce both ROS level and the extent of lipid oxidation in H_2_O_2_-induced NIH3T3 cells [104]. Cafestol and kahweol protected mouse embryonic fibroblasts from oxidative stress/toxicity triggered by electrophile acrolein through mediating Nrf2 nuclear translocation [107]. Pretreatment of mice with kahweol and cafestol prior to carbon tetrachloride exposure effectively increased cellular GSH concentration and reduced lipid peroxidation, two important indices of the prevention of ROS-induced cellular damage [103].

## 5. Antioxidant Activity of Coffee in Human Studies

The antioxidant potential of coffee derived from chemical-based antioxidant activity assays, cellular-based antioxidant activity assays and animal models have stimulated parallel investigation that has focused on determining the influence of coffee consumption on oxidative status in healthy humans and humans with diseases associated with oxidative stress.

Antioxidant activity measured in plasma of subjects both before and 1 and 2 h after a 200-mL drink of brewed coffee exhibited significantly higher antioxidant capacity than controls [108]. Similarly, 36 healthy human subjects that consumed instant coffee (800 mL/day for five days), co-extracted from green and roasted beans, respectively, also showed a significant decline in the level of lipid peroxidation (e.g., 8-isoprostaglandin F2α) in urine, which corresponded to an increase in the level of serum antioxidant enzymes (glutathione peroxidase and glutathione-S-transferase) [109]. In a controlled intervention trial in 38 participants using a cross-over design, subjects consuming 800 mL of paper filtered coffee per day over five days showed a decreased extent of DNA-damage in peripheral lymphocytes. These subjects, however, did not show similar responses in related biochemical parameters of redox status (e.g., MDA, 3-nitrotyrosine, total antioxidant levels in plasma, glutathione concentrations in blood and 8-isoprostaglandin F2α in urine) [110]. Two other recent clinic trials have also demonstrated that coffee consumption is associated with lower levels of oxidative DNA damage in both healthy subjects [111] and subjects with chronic hepatitis C [112] using 8-hydroxydeoxyguanosine as a biomarker of DNA damage. Another trial, conducted in 18 healthy male participants following consumption of a blended green and roasted coffee (750 mL in three servings every day for four weeks) showed increased activity of the Nrf2 pathway, which was defined by a significant increase in Nrf2 gene transcription in 36% of participants after four weeks of coffee consumption, in comparison to the first wash out period. This different response was affected by the Nrf2 genotype [113]. The Nrf2-activating properties of brewed coffee prepared from blended green and roasted coffee beans has been demonstrated in a pilot human intervention study using 29 healthy humans, where a daily consumption of 750 mL of brewed coffee for four weeks increased Nrf2 transcription in peripheral blood lymphocytes [114].

Inflammation is accompanied with increased oxidative stress, and the effect of coffee on reducing inflammatory-related biomarkers has shown anti-oxidative activity that paralleled reduced inflammation. For example, 47 subjects exhibiting subclinical inflammation and consuming coffee (e.g., 600 mL/day for one month and 1,200 mL/day for another month) exhibited decreased levels of interleukin 18, an inflammatory biomarker, and 8-isoprotane, a free lipid peroxidation product [115]. Subjects with a high-fat diet, known to promote oxidative stress, did not show any effect of consuming 480 mL coffee after a high-fat milk shake, as measured by changes in MDA, H_2_O_2_ or triglyceride levels in blood. These authors concluded that acute coffee consumption has no impact on postprandial oxidative stress triggered by a high-fat diet [116].

Table 3 is a summary of the important human clinical studies that have evaluated the effect of brewed coffee in modulating oxidative stress. The controversial results obtained from different studies are likely due to several reasons. Firstly, the coffee samples consumed by subjects had distinct differences in chemical composition, due to different varieties of beans, roasting temperatures and brewing methods. Secondly, it appears that different dosages and lengths of the coffee consumption treatment period also contributed to different outcomes. Finally, the biomarkers used to indicate oxidative and inflammatory status vary among different studies, which, when taken together, makes it very difficult to compare the results obtained from different human clinical trials.

**Table 3:** Summary of clinic studies evaluating the effects of coffee consumption on modulating oxidative status in healthy subjects or subjects with diseases associated with oxidative stress.

Participants and Exclusion Criteria	Experiment Design and Treatment Conditions	Indicators/Biomarkers of Oxidative Status	Results and Conclusion	Ref.
N = not given; Gender: not given; Age: not given; Eligibility: healthy, non-smoking, moderate coffee drinker; avoid antioxidant supplements and have a diet low in “coffee, wine, chocolate, tea, fruit and vegetable” in the two days before the experiment.	**Experiment group**: 200 mL of coffee (60 g of roasted ground coffee beans were prepared by a 5-min infusion in 1 L of boiling water); **Control**: 200 mL of tea.	Plasma antioxidant activity; plasma SH groups	**Results**: Experiment group: significantly ↑ plasma antioxidant capacity measured by TRAP method; the plasma SH group did not change significantly. Control: Non-significant change in both parameters. **Conclusions**: Coffee consumption improves antioxidant capacity *in vivo*.	[108]
N = 36; Gender: both; Age: 27 ± 8; Eligibility: healthy, non-smoking adults with BMI 20–25, no intake of drugs and supplements four week prior the study, no pregnancy and no blood withdrawal three weeks before the study.	**Experiment group**: instant coffee (800 mL/day for five days) co-extracted from green and roasted beans **Control**: 800 mL water/day for five days	8-Isoprostaglandin F2α; DNA migration; MDA; GPx; GST; SOD; intracellular ROS	**Results**: Experiment group showed a 15.3% 8-isoprostaglandin F2α decrease in urine and 16.1% 3-nitrotyrosine compared to the control group. Other parameters did not change significantly compared to the control. **Conclusion**: Coffee consumption protects humans against oxidative damage.	[109]
N = 38; Gender: 14 males and 24 females Age: 27.6 ± 8.0 Eligibility: healthy, non-smokers, no intake of pharmaceutical drugs, no intake of food supplements four weeks prior and during the study, no pregnancy, compliance with the protocol, no blood withdrawal three weeks before the study.	**Cross-over design**: participants were allocated into two groups (18 coffee/water and 20 water/coffee); the coffee/water group drank 800 mL coffee/day for five days, after a five-week washout phase and a one-week restriction (800 mL water/day); the water/coffee group followed the reverse order	Oxidized purines, MDA, 3-nitrotyrosine, glutathione, intracellular ROS, SOD and GPx, 8-isoprostaglandin F2α	**Results**: Coffee intake decreased DNA-damage (oxidized purines) by 12.3%; coffee intake did not markedly alter other redox status parameters. **Conclusion**: Coffee consumption prevents endogenous formation of oxidative DNA-damage in humans.	[110]
N = 40; Gender: not given Age: not given Eligibility: biopsy-proven HCV-related chronic hepatitis or cirrhosis, no consumption of other caffeine-containing beverages	**Cross-over design:** participants were allocated into Groups 1 and 2: Group 1 drank four cups coffee/day for one month and had the first blood sample taken, then continued with no coffee for 30 days and had the second blood sample taken). Group 2 followed the reverse order.	Makers of oxidative damage: 8-hydroxydeoxyguanosine, nitric oxide, advanced oxidation protein products	**Results:** 8-hydroxydeoxyguanosine was significantly lower than during abstinence. **Conclusion**: In chronic hepatitis C patients, coffee consumption induces a reduction in oxidative damage.	[112]
N = 18; Gender: male; Age: not given; Eligibility: non-smoking, BMI < 32, not on medication and does not have chronic disease, regular coffee drinker, restricted intake of coffee, caffeinated products, dietary supplements and foods rich in polyphenols.	**Intervention trial**: Four-week wash out period, four weeks of brewed coffee (prepared from a blend of green and roasted beans; 29.5 g ground coffee in 600 mL water) consumption (750 mL/day); and another eight weeks of wash out.	Nrf2 gene transcription level in blood sample	**Results**: Thirty six percent of participants displayed a significant >1.5 alteration of Nrf2 transcription after coffee consumption compared to the wash out period; 64% of the participants showed no change. **Conclusion** : Induction of Nrf2 gene transcription by coffee in humans depends on the genotype of subjects.	[113]	
N = 29; Gender: not given; Age: not given; Eligibility: healthy volunteers	**Intervention trial**: Four-week wash out period, four weeks of brewed coffee (prepared from a blend of green and roasted beans; 29.5 g ground coffee in 600 mL water) consumption (750 mL/day); and another four weeks of wash out.	Nrf2 transcription level in peripheral blood lymphocytes	**Results:** Coffee consumption increased Nrf2 transcription in peripheral blood lymphocytes on average. **Conclusion**: Coffee acts as a modulator of Nrf2-depended gene response in humans, but genetic polymorphisms play an important role in the individual response pattern.	[114]
N = 47; Gender: 11 male and 36 female; Age: 54 ± 9; Eligibility: habitual coffee drinkers, free of type 2 diabetes.	**Single blind, three-stage clinical trial**: One-month wash out period; 600 mL filtered coffee per day for one month, followed by 1,200 mL filtered coffee per day for another month.	IL-18, IL-6, macrophage migration inhibitory factor, leptin, C-reactive protein, serum amyloid A, 8-isoprostane, nitrotyrosine, LDL:HDL cholesterol	**Results**: Significant ↓ in IL-18, 8-isoprostane, LDL:HDL cholesterol. **Conclusion**: Coffee consumption appears to have beneficial effects on subclinical inflammation.	[115]	
N = 16; Gender: 8 male and 8 female; Age: 29.2 ± 14.4; Eligibility: non-smoking, healthy, without a history of cardiovascular or metabolic disease, did not subject use medications or dietary supplements throughout the study period.	**Random, crossover design:** Each subject has three visits (one visit every day); at each visit, the subject firstly consumes a milk shake (containing 12.2 kCal/kg of body weight) followed by consuming either 480 mL of freshly brewed caffeinated coffee, or decaffeinated coffee, or water. The order of assignment for the three days of testing was random; each subject received all three conditions over the course of the study.	MDA, H_2_O_2_, triglycerides	**Results**: Coffee had no impact on MDA, H_2_O_2_ or triglyceride level in blood. **Conclusion**: Acute coffee consumption following a high-fat milk shake has no impact on postprandial oxidative stress.	[116]	

## 6. Conclusions

Coffee represents a very popular beverage consumed by adults, globally, for its desirable organoleptic qualities, noted stimulant effects for many consumers and, more recently, reported potential health benefits. Indeed, in the last decade, many studies have reported coffee consumption to be associated with a reduced risk of several chronic diseases; the physiological chemistry that underlies these claims often involves a number of bioactive compounds that exhibit antioxidant and anti-peroxidation activities, respectively. This review has focused on describing many of the *in vitro* chemical antioxidant assays used to assess the antioxidant capacity of brewed coffee and its related individual constituents. These tests have enabled characterization of different mechanisms of antioxidant action that are germane to many bioactive components in this complex beverage. However, chemical assays do not enable the discovery of evidence for cellular and molecular defenses that are involved in detoxifying ROS. Moreover, the complexity of determining the antioxidant capacity of brewed coffee strongly suggests that a battery of tests that define distinct mechanisms of action is required. There is also a requirement for advancement in both assay development and the selection of biochemical and genomic end-point parameters that can assist in developing more conclusive decisions on the health benefits of coffee and its constituents. To this end, specific cell-based antioxidant model systems have enabled the demonstration of coffee components, both naturally present and derived from the roasting process, to enhance the redox potential of isolated cells and the activation of antioxidant-response elements attributed to oxidative stress. These specific findings have proven to be complementary to *in vivo* oxidative/antioxidant balances studies conducted in both animal and human subjects.

## References

[1] Gunalan G., Myla N., Balabhaskar R. (2012). *In vitro* antioxidant analysis of selected coffee bean varieities. J. Chem. Pharm. Res..

[2] Fogliano V., Morales F.J. (2011). Estimation of dietary intake of melanoidins from coffee and bread. Food Funct..

[3] Reichardt N., Gniechwitz D., Steinhart H., Bunzel M., Blaut M. (2009). Characterization of high molecular weight coffee fractions and their fermentation by human intestinal microbiota. Mol. Nutr. Food Res..

[4] Morales F.J., Jiménez-pérez S. (2004). Peroxyl radical scavenging activity of melanoidins in aqueous systems. Eur. Food Res. Technol..

[5] Mader E.A., Davidson E.R., Mayer J.M. (2007). Large ground-state entropy changes for hydrogen atom transfer reactions of iron complexes. J. Am. Chem. Soc..

[6] Ashby E.C. (1988). Single-electron transfer, a major reaction pathway in organic chemistry. An answer to recent criticisms. Acc. Chem. Res..

[7] Shalaby E.A., Shanab S.M.M. (2013). Antioxidant compounds, assays of determination and mode of action. Afr. J. Pharm. Pharmacol..

[8] Ramalakshmi K., Rahath Kubra I., Jagan Mohan Rao L. (2008). Antioxidant potential of low-grade coffee beans. Food Res. Int..

[9] Anese M., Nicoli M.C. (2003). Antioxidant properties of ready-to-drink coffee brews. J. Agric. Food Chem..

[10] Madhava Naidu M., Sulochanamma G., Sampathu S.R., Srinivas P. (2008). Studies on extraction and antioxidant potential of green coffee. Food Chem..

[11] Ludwig I.A., Sanchez L., Caemmerer B., Kroh L.W., de Peña M.P., Cid C. (2012). Extraction of coffee antioxidants: Impact of brewing time and method. Food Res. Int..

[12] Cammerer B., Kroh L.W. (2006). Antioxidant activity of coffee brew. Eur. Food Res. Technol..

[13] Sacchetti G., di Mattia C., Pittia P., Mastrocola D. (2009). Effect of roasting degree, equivalent thermal effect and coffee type on the radical scavenging activity of coffee brews and their phenolic fraction. J. Food Eng..

[14] Del Castillo M.D., Ames J.M., Gordon M.H. (2002). Effect of roasting on the antioxidant activity of coffee brews. J. Agric. Food Chem..

[15] Sánchez-González I., Jiménez-Escrig A., Saura-Calixto F. (2005). *In vitro* antioxidant activity of coffees brewed using different procedures (Italian, espresso and filter). Food Chem..

[16] Chu Y.-F., Brown P.H., Lyle B.J., Chen Y., Black R.M., Williams C.E., Lin Y.-C., Hsu C.-W., Cheng I.H. (2009). Roasted Coffees High in Lipophilic Antioxidants and Chlorogenic Acid Lactones Are More Neuroprotective than Green Coffees. J. Agric. Food Chem..

[17] Kwon D.Y., Choi K.H., Kim S.J., Choi D.W., Kim Y.S., Kim Y.C. (2005). Peroxyl radical-scavenging activity of coffee brews. Eur. Food Res. Technol..

[18] Mullen W., Nemzer B., Ou B., Stalmach A., Hunter J., Clifford M.N., Combet E. (2011). The antioxidant and chlorogenic acid profiles of whole coffee fruits are influenced by the extraction procedures. J. Agric. Food Chem..

[19] Brezová V., Šlebodová A., Staško A. (2009). Coffee as a source of antioxidants: An EPR study. Food Chem.

[20] Parras P., Martínez-Tomé M., Jiménez A.M., Murcia M.A. (2007). Antioxidant capacity of coffees of several origins brewed following three different procedures. Food Chem..

[21] Kedare S.B., Singh R.P. (2011). Genesis and development of DPPH method of antioxidant assay. J. Food Sci. Technol..

[22] Blois M.S. (1958). Antioxidant Determinations by the Use of a Stable Free Radical. Nature.

[23] Prior R.L., Wu X., Schaich K. (2005). Standardized methods for the determination of antioxidant capacity and phenolics in foods and dietary supplements. J. Agric. Food Chem..

[24] Re R., Pellegrini N., Proteggente A., Pannala A., Yang M., Rice-Evans C. (1999). Antioxidant activity applying an improved ABTS radical cation decolorization assay. Free Rad. Biol. Med..

[25] Gil M.I., Tomas-Barberan F.A., Hess-Pierce B., Holcroft D.M., Kader A.A. (2000). Antioxidant activity of pomegranate juice and its relationship with phenolic composition and processing. J. Agric. Food Chem..

[26] Antonio C. (1998). An end-point method for estimation of the total antioxidant activity in plant material. Phytochem. Anal..

[27] Leong L.P., Shui G. (2002). An investigation of antioxidant capacity of fruits in Singapore markets. Food Chem..

[28] Shalaby E.A., Shanab S.M.M. (2013). Comparison of DPPH and ABTS assays for determing antioxidant potential of water and methanol extracts of spirulina platensis. Indian J. Geo-Mar. Sci..

[29] Floegel A., Kim D.-O., Chung S.-J., Koo S.I., Chun O.K. (2011). Comparison of ABTS/DPPH assays to measure antioxidant capacity in popular antioxidant-rich US foods. J. Food Compos. Anal..

[30] Bravo J., Monente C., Juániz I., de Peña M.P., Cid C. (2013). Influence of extraction process on antioxidant capacity of spent coffee. Food Res. Int..

[31] Vignoli J.A., Bassoli D.G., Benassi M.T. (2011). Antioxidant activity, polyphenols, caffeine and melanoidins in soluble coffee: The influence of processing conditions and raw material. Food Chem..

[32] Svilaas A., Sakhi A.K., Andersen L.F., Svilaas T., Strom E.C., Jacobs D.R., Ose L., Blomhoff R. (2004). Intakes of antioxidants in coffee, wine, and vegetables are correlated with plasma carotenoids in humans. J. Nutr..

[33] Yanagimoto K., Lee K.-G., Ochi H., Shibamoto T. (2002). Antioxidative activity of heterocyclic compounds found in coffee volatiles produced by maillard reaction. J. Agric. Food Chem..

[34] Benzie I.F.F., Strain J.J. (1996). The Ferric Reducing Ability of Plasma (FRAP) as a Measure of “Antioxidant Power”: The FRAP Assay. Anal. Biochem..

[35] Valadao V.S.J., Queiroz Y.S., Gotlieb S.L.D., da Silva Torres E.A.F. (2014). Stability of phenolic compounds and antioxidant capacity of regular and decaffeinated coffees. Braz. Arch. Biol. Technol..

[36] Wayner D.D., Burton G.W., Ingold K.U., Barclay L.R., Locke S.J. (1987). The relative contributions of vitamin E, urate, ascorbate and proteins to the total peroxyl radical-trapping antioxidant activity of human blood plasma. Biochim. Biophys. Acta.

[37] Cao G., Alessio H.M., Cutler R.G. (1993). Oxygen-radical absorbance capacity assay for antioxidants. Free Radic. Biol. Med..

[38] Kitts D.D., Hu C. (2005). Biological and chemical assessment of antioxidant activity of sugar-lysine model maillard reaction products. Ann. N. Y. Acad. Sci..

[39] Liu Y., Kitts D.D. (2012). Activation of antioxidant response element (ARE)-dependent genes by roasted coffee extracts. Food Funct..

[40] Ou B., Hampsch-Woodill M., Prior R.L. (2001). Development and Validation of an Improved Oxygen Radical Absorbance Capacity Assay Using Fluorescein as the Fluorescent Probe. J. Agric. Food Chem..

[41] Gómez-Ruiz J.Á., Leake D.S., Ames J.M. (2007). *In Vitro* Antioxidant Activity of Coffee Compounds and Their Metabolites. J. Agric. Food Chem..

[42] Prior R.L., Hoang H., Gu L., Wu X., Bacchiocca M., Howard L., Hampsch-Woodill M., Huang D., Ou B., Jacob R. (2003). Assays for Hydrophilic and Lipophilic Antioxidant Capacity (oxygen radical absorbance capacity (ORACFL)) of Plasma and Other Biological and Food Samples. J. Agric. Food Chem..

[43] Lloyd R.V., Hanna P.M., Mason R.P. (1997). The origin of the hydroxyl radical oxygen in the Fenton reaction. Free Radic. Biol. Med..

[44] Klein S.M., Cohen G., Cederbaum A.I. (1981). Production of formaldehyde during metabolism of dimethyl sulfoxide by hydroxyl radical generating systems. Biochemistry.

[45] Gutteridge J.M. (1987). Ferrous-salt-promoted damage to deoxyribose and benzoate. The increased effectiveness of hydroxyl-radical scavengers in the presence of EDTA. Biochem. J..

[46] Althaus J., Andrus P., Williams C., VonVoigtlander P., Cazers A., Hall E. (1993). The use of salicylate hydroxylation to detect hydroxyl radical generation in ischemic and traumatic brain injury. Mol. Chem. Neuropathol..

[47] Wijewickreme A.N., Kitts D.D. (1998). Modulation of metal-induced genotoxicity by Maillard reaction products isolated from coffee. Food Chem. Toxicol..

[48] Li L., Abe Y., Kanagawa K., Usui N., Imai K., Mashino T., Mochizuki M., Miyata N. (2004). Distinguishing the 5,5-dimethyl-1-pyrroline N-oxide (DMPO)-OH radical quenching effect from the hydroxyl radical scavenging effect in the ESR spin-trapping method. Anal. Chim. Acta.

[49] Chung H.Y., Baek B.S., Song S.H., Kim M.S., Huh J.I., Shim K.H., Kim K.W., Lee K.H. (1997). Xanthine dehydrogenase/xanthine oxidase and oxidative stress. Age.

[50] Ewing J.F., Janero D.R. (1995). Microplate superoxide dismutase assay employing a nonenzymatic superoxide generator. Anal. Biochem..

[51] Fox G.P., Wu A., Yiran L., Force L. (2013). Variation in caffeine concentration in single coffee beans. J. Agric. Food Chem..

[52] Casal S., Beatriz Oliveira M., Ferreira M.A. (2000). HPLC/diode-array applied to the thermal degradation of trigonelline, nicotinic acid and caffeine in coffee. Food Chem..

[53] Tfouni S.A.V., Carreiro L.B., Teles C.R.A., Furlani R.P.Z., Cipolli K.M.V.A.B., Camargo M.C.R. (2014). Caffeine and chlorogenic acids intake from coffee brew: Influence of roasting degree and brewing procedure. Int. J. Food Sci. Technol..

[54] McCusker R.R., Fuehrlein B., Goldberger B.A., Gold M.S., Cone E.J. (2006). Caffeine content of decaffeinated coffee. J. Anal. Toxicol..

[55] León-Carmona J.R., Galano A. (2011). Is Caffeine a Good Scavenger of Oxygenated Free Radicals?. J.Phys. Chem. B.

[56] Shi X., Dalal N.S., Jain A.C. (1991). Antioxidant behaviour of caffeine: Efficient scavenging of hydroxyl radicals. Food Chem. Toxicol..

[57] Kumar S.S., Devasagayam T.P., Jayashree B., Kesavan P.C. (2001). Mechanism of protection against radiation-induced DNA damage in plasmid pBR322 by caffeine. Int. J. Radiat. Biol..

[58] Lee C. (2000). Antioxidant ability of caffeine and its metabolites based on the study of oxygen radical absorbing capacity and inhibition of LDL peroxidation. Clin. Chim. Acta.

[59] Devasagayam T.P.A., Kamat J.P., Mohan H., Kesavan P.C. (1282). Caffeine as an antioxidant: Inhibition of lipid peroxidation induced by reactive oxygen species. Biochim. Biophys. Acta.

[60] Stalmach A., Steiling H., Williamson G., Crozier A. (2010). Bioavailability of chlorogenic acids following acute ingestion of coffee by humans with an ileostomy. Arch. Biochem. Biophys..

[61] Clifford M.N. (1999). Chlorogenic acids and other cinnamates-nature, occurrence and dietary burden. J. Sci. Food Agric..

[62] Perrone D., Farah A., Donangelo C.M., de Paulis T., Martin P.R. (2008). Comprehensive analysis of major and minor chlorogenic acids and lactones in economically relevant Brazilian coffee cultivars. Food Chem..

[63] Zang L.Y., Cosma G., Gardner H., Castranova V., Vallyathan V. (2003). Effect of chlorogenic acid on hydroxyl radical. Mol. Cell. Biochem..

[64] Kweon M.H., Hwang H.J., Sung H.C. (2001). Identification and antioxidant activity of novel chlorogenic acid derivatives from bamboo (Phyllostachys edulis). J. Agric. Food Chem..

[65] Magadula J.J., Tewtrakul S., Gatto J., Richomme P. (2011). *In vitro* antioxidant and anti-HIV-1 protease (PR) activities of two Clusiaceae plants endemic to Tanzania. Int. J. Biol. Chem. Sci..

[66] Xu J.G., Hu Q.P., Liu Y. (2012). Antioxidant and DNA-protective activities of chlorogenic acid isomers. J. Agric. Food Chem..

[67] Namiki M. (1988). Chemistry of Maillard reactions: Recent studies on the browning reaction mechanism and the development of antioxidants and mutagens. Adv. Food Res..

[68] Rufián-Henares J.A., Morales F.J. (2007). Functional properties of melanoidins: *In vitro* antioxidant, antimicrobial and antihypertensive activities. Food Res. Int..

[69] Liu Y., Kitts D.D. (2011). Confirmation that the Maillard reaction is the principle contributor to the antioxidant capacity of coffee brews. Food Res. Int..

[70] Borrelli R.C., Visconti A., Mennella C., Anese M., Fogliano V. (2002). Chemical characterization and antioxidant properties of coffee melanoidins. J. Agric. Food Chem..

[71] Gniechwitz D., Reichardt N., Ralph J., Blaut M., Steinhart H., Bunzel M. (2008). Isolation and characterisation of a coffee melanoidin fraction. J. Sci. Food Agric..

[72] Moreira A.S., Nunes F.M., Domingues M.R., Coimbra M.A. (2012). Coffee melanoidins: Structures, mechanisms of formation and potential health impacts. Food Funct..

[73] Delgado-Andrade C., Rufian-Henares J.A., Morales F.J. (2005). Assessing the antioxidant activity of melanoidins from coffee brews by different antioxidant methods. J. Agric. Food Chem..

[74] Morales F.J. (2005). Assessing the non-specific hydroxyl radical scavenging properties of melanoidins in a Fenton-type reaction system. Anal. Chim. Acta.

[75] Delgado-Andrade C., Morales F.J. (2005). Unraveling the contribution of melanoidins to the antioxidant activity of coffee brews. J. Agric. Food Chem..

[76] Rufian-Henares J.A., Morales F.J. (2007). Effect of *in vitro* enzymatic digestion on antioxidant activity of coffee melanoidins and fractions. J. Agric. Food Chem..

[77] Samunl A., Neta P. (1973). Electron spin resonance study of the reaction of hydroxyl radicals with pyrrole, imidazole, and related compound. J. Phys. Chem..

[78] Zhang D., Xie L., Wei Y., Liu Y., Jia G., Zhou F., Ji B. (2013). Development of a cell-based antioxidant activity assay using dietary fatty acid as oxidative stressor. Food Chem..

[79] Sessa M., Tsao R., Liu R., Ferrari G., Donsi F. (2011). Evaluation of the stability and antioxidant activity of nanoencapsulated resveratrol during *in vitro* digestion. J. Agric. Food Chem..

[80] Wolfe K.L., Liu R.H. (2007). Cellular antioxidant activity (CAA) assay for assessing antioxidants, foods, and dietary supplements. J. Agric. Food Chem..

[81] Ziberna L., Lunder M., Moze S., Vanzo A., Tramer F., Passamonti S., Drevensek G. (2010). Acute cardioprotective and cardiotoxic effects of bilberry anthocyanins in ischemia-reperfusion injury: Beyond concentration-dependent antioxidant activity. Cardiovasc. Toxicol..

[82] Roy M.K., Juneja L.R., Isobe S., Tsushida T. (2009). Steam processed broccoli (Brassica oleracea) has higher antioxidant activity in chemical and cellular assay systems. Food Chem..

[83] Cooke M.S., Evans M.D., Dizdaroglu M., Lunec J. (2003). Oxidative DNA damage: Mechanisms, mutation, and disease. FASEB J..

[84] Motohashi H., Yamamoto M. (2004). Nrf2-Keap1 defines a physiologically important stress response mechanism. Trends Mol. Med..

[85] Boettler U., Sommerfeld K., Volz N., Pahlke G., Teller N., Somoza V., Lang R., Hofmann T., Marko D. (2011). Coffee constituents as modulators of Nrf2 nuclear translocation and ARE (EpRE)-dependent gene expression. J. Nutr. Biochem..

[86] Tiwari K.K., Chu C., Couroucli X., Moorthy B., Lingappan K. (2014). Differential concentration-specific effects of caffeine on cell viability, oxidative stress, and cell cycle in pulmonary oxygen toxicity *in vitro*. Biochem. Biophys. Res. Commun..

[87] Jagdeo J., Brody N. (2011). Complementary antioxidant function of caffeine and green tea polyphenols in normal human skin fibroblasts. J. Drugs Dermatol..

[88] Abreu R.V., Silva-Oliveira E.M., Moraes M.F., Pereira G.S., Moraes-Santos T. (2011). Chronic coffee and caffeine ingestion effects on the cognitive function and antioxidant system of rat brains. Pharmacol. Biochem. Behav..

[89] Lv X., Chen Z., Li J., Zhang L., Liu H., Huang C., Zhu P. (2010). Caffeine protects against alcoholic liver injury by attenuating inflammatory response and oxidative stress. Inflamm. Res..

[90] Prasanthi J.R., Dasari B., Marwarha G., Larson T., Chen X., Geiger J.D., Ghribi O. (2010). Caffeine protects against oxidative stress and Alzheimer’s disease-like pathology in rabbit hippocampus induced by cholesterol-enriched diet. Free Radic. Biol. Med..

[91] Cha J.W., Piao M.J., Kim K.C., Yao C.W., Zheng J., Kim S.M., Hyun C.L., Ahn Y.S., Hyun J.W. (2014). The Polyphenol Chlorogenic Acid Attenuates UVB-mediated Oxidative Stress in Human HaCaT Keratinocytes. Biomol. Ther..

[92] Li S., Bian H., Liu Z., Wang Y., Dai J., He W., Liao X., Liu R., Luo J. (2012). Chlorogenic acid protects MSCs against oxidative stress by altering FOXO family genes and activating intrinsic pathway. Eur. J. Pharmacol..

[93] Pavlica S., Gebhardt R. (2005). Protective effects of ellagic and chlorogenic acids against oxidative stress in PC12 cells. Free Radic. Res..

[94] Baeza G., Amigo-Benavent M., Sarriá B., Goya L., Mateos R., Bravo L. (2014). Green coffee hydroxycinnamic acids but not caffeine protect human HepG2 cells against oxidative stress. Food Res. Int..

[95] Boettler U., Volz N., Pahlke G., Teller N., Kotyczka C., Somoza V., Stiebitz H., Bytof G., Lantz I., Lang R. (2011). Coffees rich in chlorogenic acid or N-methylpyridinium induce chemopreventive phase II-enzymes via the Nrf2/ARE pathway *in vitro* and *in vivo*. Mol. Nutr. Food Res..

[96] Tsuchiya T., Suzuki O., Igarashi K. (1996). Protective effects of chlorogenic acid on paraquat-induced oxidative stress in rats. Biosci. Biotechnol. Biochem..

[97] Amjid A., Pawan K. (2013). Protective effect of chlorogenic acid against diabetic nephropathy in high fat diet/streptozotocin induced type-2 diabetic rats. Int. J. Pharm. Pharm. Sci..

[98] Goya L., Delgado-Andrade C., Rufian-Henares J.A., Bravo L., Morales F.J. (2007). Effect of coffee melanoidin on human hepatoma HepG2 cells. Protection against oxidative stress induced by tert-butylhydroperoxide. Mol. Nutr. Food Res..

[99] Vitaglione P., Morisco F., Mazzone G., Amoruso D.C., Ribecco M.T., Romano A., Fogliano V., Caporaso N., D’Argenio G. (2010). Coffee reduces liver damage in a rat model of steatohepatitis: The underlying mechanisms and the role of polyphenols and melanoidins. Hepatology.

[100] Daglia M., Racchi M., Papetti A., Lanni C., Govoni S., Gazzani G. (2004). *In vitro* and *ex vivo* antihydroxyl radical activity of green and roasted coffee. J. Agric. Food Chem..

[101] Zhou J., Zhou S., Zeng S. (2013). Experimental diabetes treated with trigonelline: Effect on beta cell and pancreatic oxidative parameters. Fundam. Clin. Pharmacol..

[102] Hwang Y.P., Jeong H.G. (2008). The coffee diterpene kahweol induces heme oxygenase-1 via the PI3K and p38/Nrf2 pathway to protect human dopaminergic neurons from 6-hydroxydopamine-derived oxidative stress. FEBS Lett..

[103] Lee K.J., Choi J.H., Jeong H.G. (2007). Hepatoprotective and antioxidant effects of the coffee diterpenes kahweol and cafestol on carbon tetrachloride-induced liver damage in mice. Food Chem. Toxicol..

[104] Lee K.J., Jeong H.G. (2007). Protective effects of kahweol and cafestol against hydrogen peroxide-induced oxidative stress and DNA damage. Toxicol. Lett..

[105] Lang R., Wahl A., Stark T., Hofmann T. (2011). Urinary N-methylpyridinium and trigonelline as candidate dietary biomarkers of coffee consumption. Mol. Nutr. Food Res..

[106] Somoza V., Lindenmeier M., Wenzel E., Frank O., Erbersdobler H.F., Hofmann T. (2003). Activity-Guided Identification of a Chemopreventive Compound in Coffee Beverage Using *in Vitro* and *in Vivo* Techniques. J. Agric. Food Chem..

[107] Higgins L.G., Cavin C., Itoh K., Yamamoto M., Hayes J.D. (2008). Induction of cancer chemopreventive enzymes by coffee is mediated by transcription factor Nrf2. Evidence that the coffee-specific diterpenes cafestol and kahweol confer protection against acrolein. Toxicol. Appl. Pharmacol..

[108] Natella F., Nardini M., Giannetti I., Dattilo C., Scaccini C. (2002). Coffee drinking influences plasma antioxidant capacity in humans. J. Agric. Food Chem..

[109] Hoelzl C., Knasmuller S., Wagner K.H., Elbling L., Huber W., Kager N., Ferk F., Ehrlich V., Nersesyan A., Neubauer O. (2010). Instant coffee with high chlorogenic acid levels protects humans against oxidative damage of macromolecules. Mol. Nutr. Food Res..

[110] Mišík M., Hoelzl C., Wagner K.-H., Cavin C., Moser B., Kundi M., Simic T., Elbling L., Kager N., Ferk F. (2010). Impact of paper filtered coffee on oxidative DNA-damage: Results of a clinical trial. Mutat. Res..

[111] Hori A., Kasai H., Kawai K., Nanri A., Sato M., Ohta M., Mizoue T. (2014). Coffee Intake is Associated With Lower Levels of Oxidative DNA Damage and Decreasing Body Iron Storage in Healthy Women. Nutr. Cancer.

[112] Cardin R., Piciocchi M., Martines D., Scribano L., Petracco M., Farinati F. (2013). Effects of coffee consumption in chronic hepatitis C: A randomized controlled trial. Dig. Liver Dis..

[113] Boettler U., Volz N., Teller N., Haupt L.M., Bakuradze T., Eisenbrand G., Bytof G., Lantz I., Griffiths L.R., Marko D. (2012). Induction of antioxidative Nrf2 gene transcription by coffee in humans: Depending on genotype?. Mol. Biol. Rep..

[114] Volz N., Boettler U., Winkler S., Teller N., Schwarz C., Bakuradze T., Eisenbrand G., Haupt L., Griffiths L.R., Stiebitz H. (2012). Effect of coffee combining green coffee bean constituents with typical roasting products on the Nrf2/ARE pathway *in vitro* and *in vivo*. J. Agric. Food Chem..

[115] Kempf K., Herder C., Erlund I., Kolb H., Martin S., Carstensen M., Koenig W., Sundvall J., Bidel S., Kuha S. (2010). Effects of coffee consumption on subclinical inflammation and other risk factors for type 2 diabetes: A clinical trial. Am. J. Clin. Nutr..

[116] Bloomer R.J., Trepanowski J.F., Farney T.M. (2013). Influence of acute coffee consumption on postprandial oxidative stress. Nutr. Metab. Insights.

